# Supplementary figures and images for: Cysteinyl leukotriene receptor 1 is dispensable for osteoclast differentiation and bone resorption
Source: PLoS One. 2022 Nov 17;17(11):e0277307. doi: 10.1371/journal.pone.0277307 (PMC9671454; doi:10.1371/journal.pone.0277307)

S1 Fig

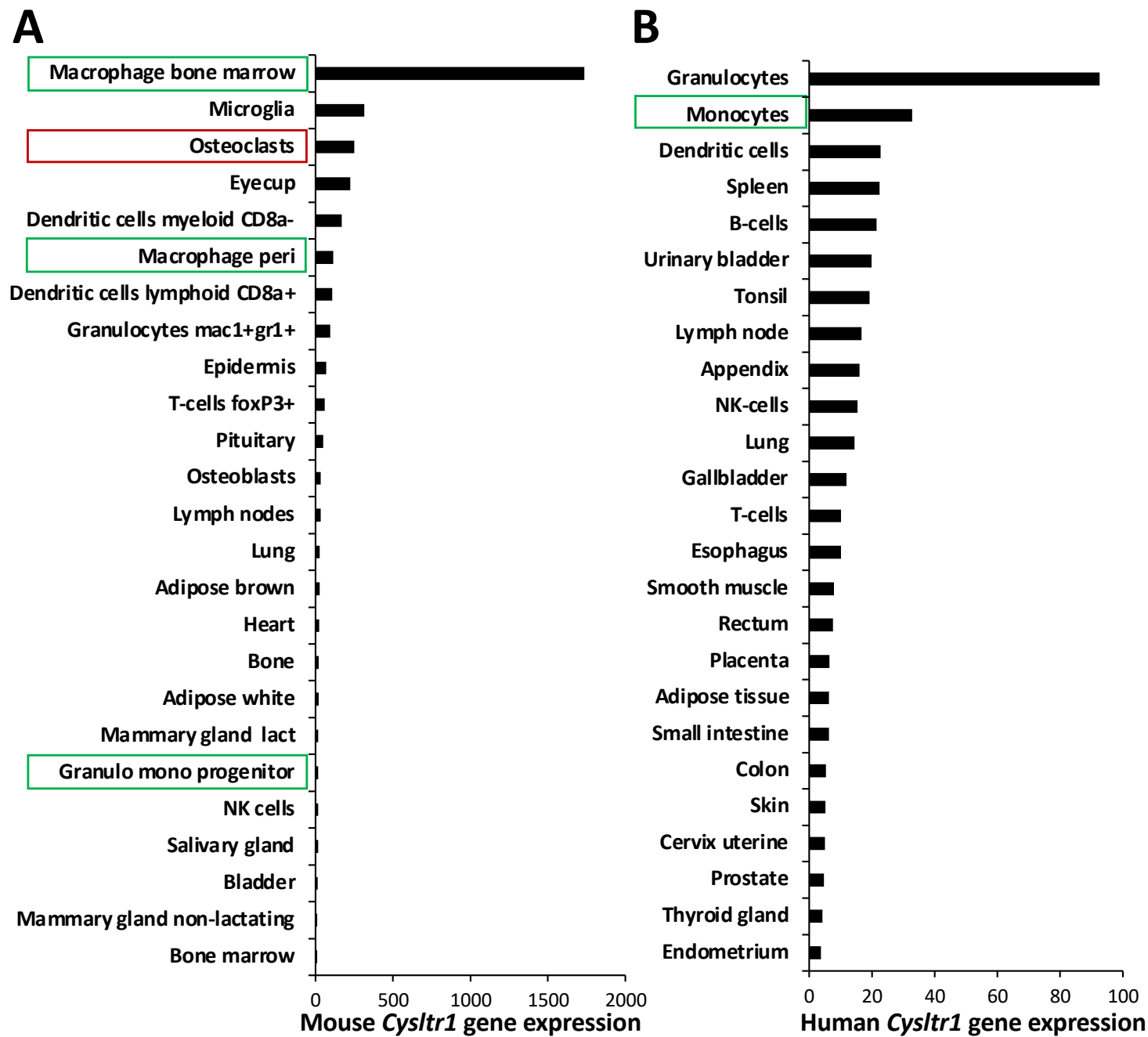

Supplement: S1 Fig — A list of the top 25 Cysltr1-expressing cells and tissues in mice (A) and humans (B). These data were obtained from the public databases BIOGPS and THE HUMAN PROTEIN ATLAS. (PDF) [file pone.0277307.s001.pdf]

**S2 Fig**

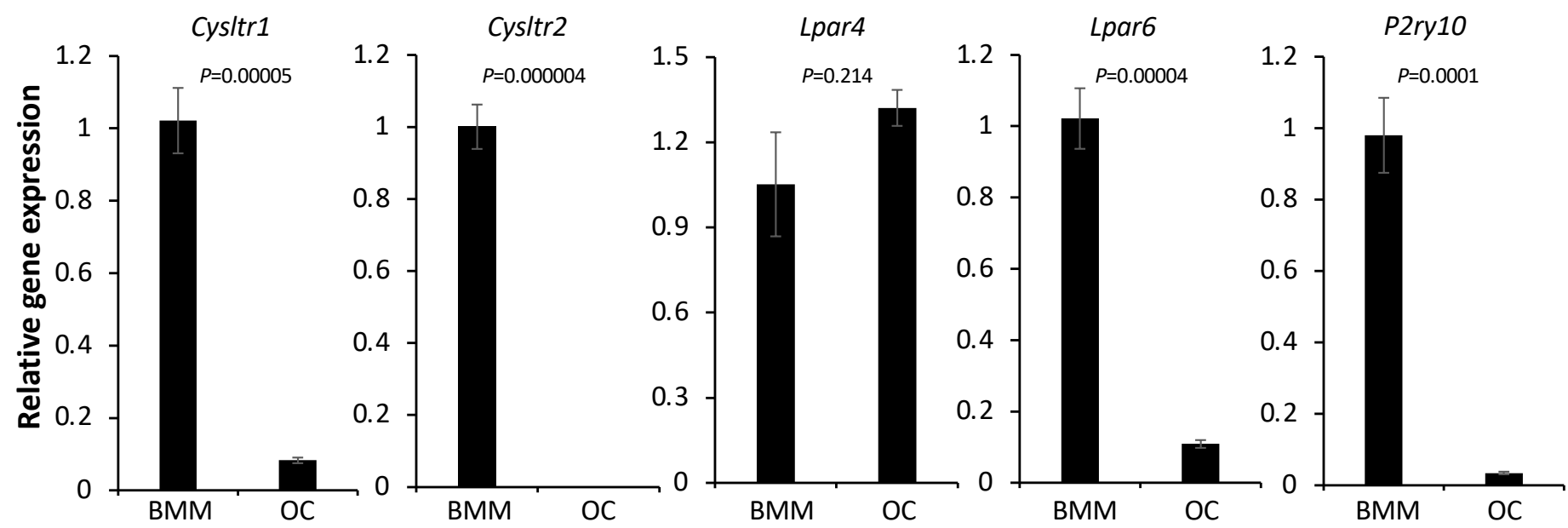

Supplement: S2 Fig — Bone marrow macrophages (BMM) were cultured with RANKL for 3 days for osteoclast (OC) formation. A qPCR analysis of Cysltr1 and its paralog mRNA expression levels in WT (C57BL/6) BMM and OC. BMM, n = 4; OC, n = 4. Gene expression was normalized with 18s ribosomal RNA (Rn18s). (PDF) [file pone.0277307.s002.pdf]

S5 Fig

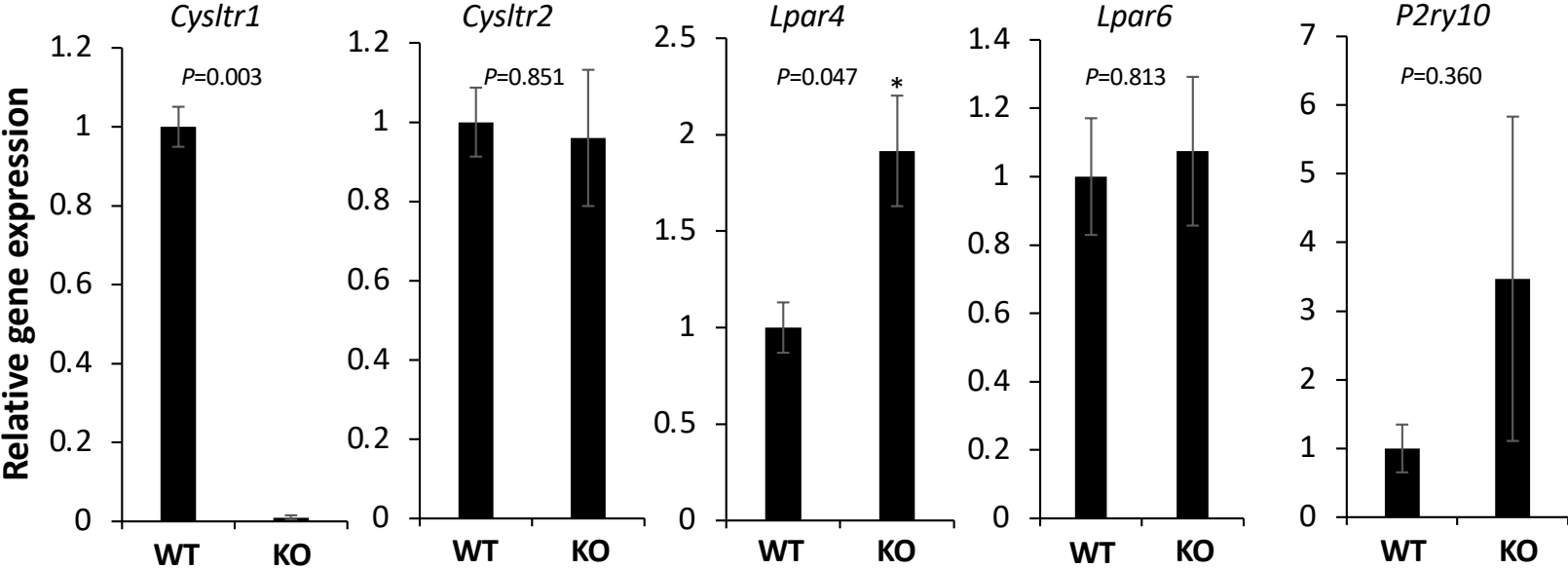

Supplement: S5 Fig — A quantitative PCR analysis was performed for Cysltr1 and its paralog mRNA expression in bone marrow macrophages from WT and Cysltr1 KO mice. WT, n = 3; KO, n = 3. Gene expression was normalized with glyceraldehyde-3-phosphate dehydrogenase (Gapdh) as a housekeeping gene. (PDF) [file pone.0277307.s005.pdf]

S6 Fig

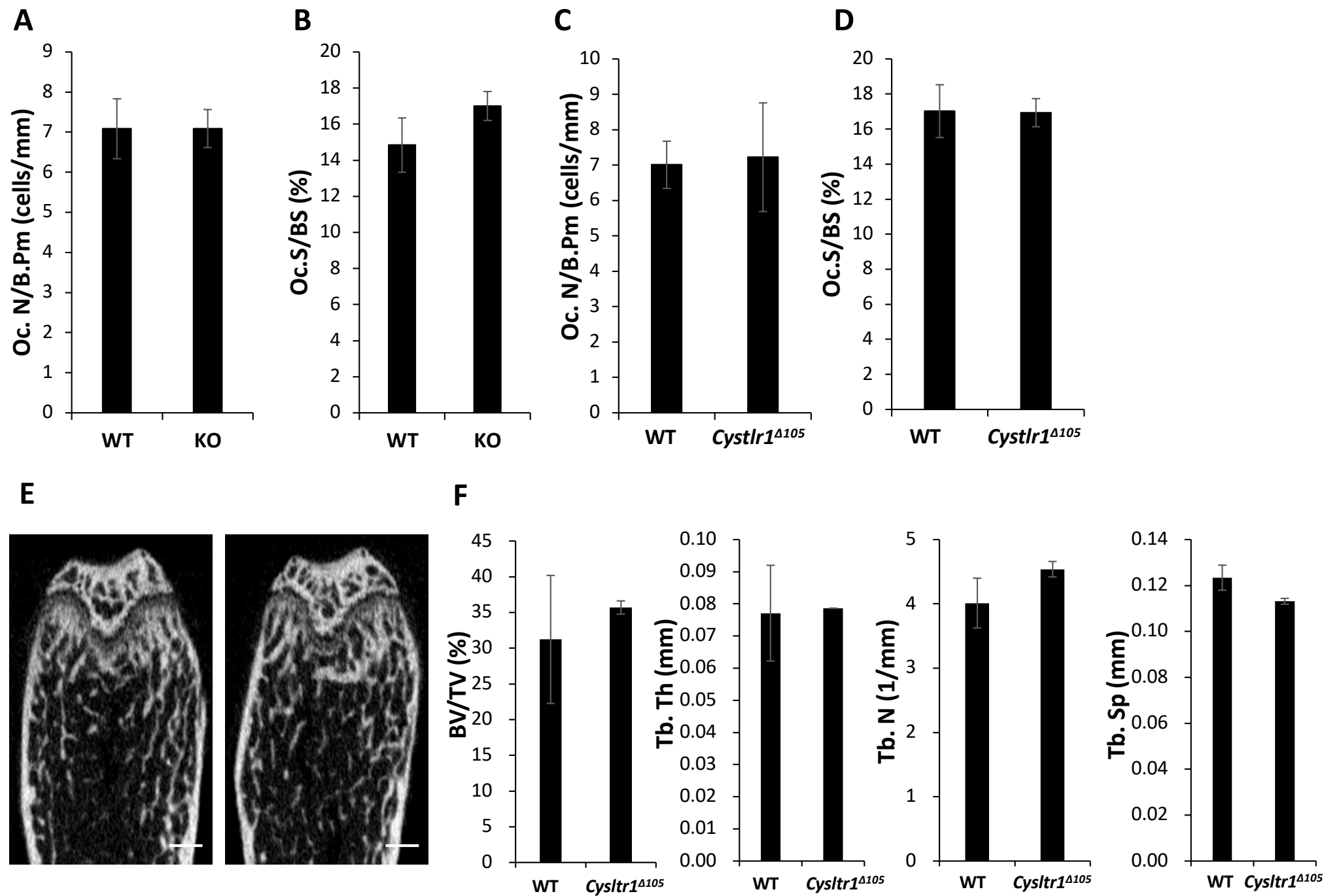

Supplement: S6 Fig — Histomorphometry of the femur from WT, Cysltr1 KO (A and B) or Cysltr1Δ105 (C and D) were analyzed using TRAP and hematoxylin staining to detect osteoclasts. Each sample, n = 2. (E) Micro-computed tomography (μCT) images of the femur of WT and Cysltr1Δ105 mice under physiological condition. Scale bar: 500 μm. (F) Trabecular bone volume per tissue volume (BV/TV), trabecular number (Tb.N), trabecular thickness (Tb.Th), and trabecular separation (Tb.Sp) in the metaphyseal region of the femur of (E). Each sample, n = 2. (PDF) [file pone.0277307.s006.pdf]
