# Supplementary material for: Cysteinyl leukotriene receptor 1 is dispensable for osteoclast differentiation and bone resorption
Source: PLoS One. 2022 Nov 17;17(11):e0277307. doi: 10.1371/journal.pone.0277307 (PMC9671454; doi:10.1371/journal.pone.0277307)

S3 Fig

A

Genotype	CysLTR1 amino acid sequence (B6;D2)	Number of AA
WT	MYLQGTKQTFLENMNGTENLTTSLINNTCHDTIDEFRNQVYSTMYSVISVVGFFGNSFVLYVLIKTYHEKSAFQ<br>VYMINLAIADLLCVCTLPLRVVYYVHKGKWLFGDFLCRLTTYALYVNLYCSIFFMTAMSFFRCVAIVFPVQNIN<br>LVTQKKARFVCIGIWIFVILTSSPFLMYKSYQDEKNNTKCFEPPQNNQAKKYVLILHYVSLFFGFIIPFVTIIV<br>CYTMIILTLLKNTMKKNMPSRRKAIGMIIVVTAAFLVSFMPYHIQRTIHLHLLHSETRPCDSVLRMQKSVVITL<br>SLAASNCCFDPLLYFFSGGNFRRRLSTFRKHSLSSMTYVPKKKASLPEKGEEICNE	352
KO	MYLQGTKQTFLENMNGTENLTTSLINNTCHDTIDEFRNQVYSTMYSVISVVGFFGNSFVLYVLIKTYHEKSAFQ<br>VYMINLAILTSIGASSL*	91

Unintended sequence by frame shift

B

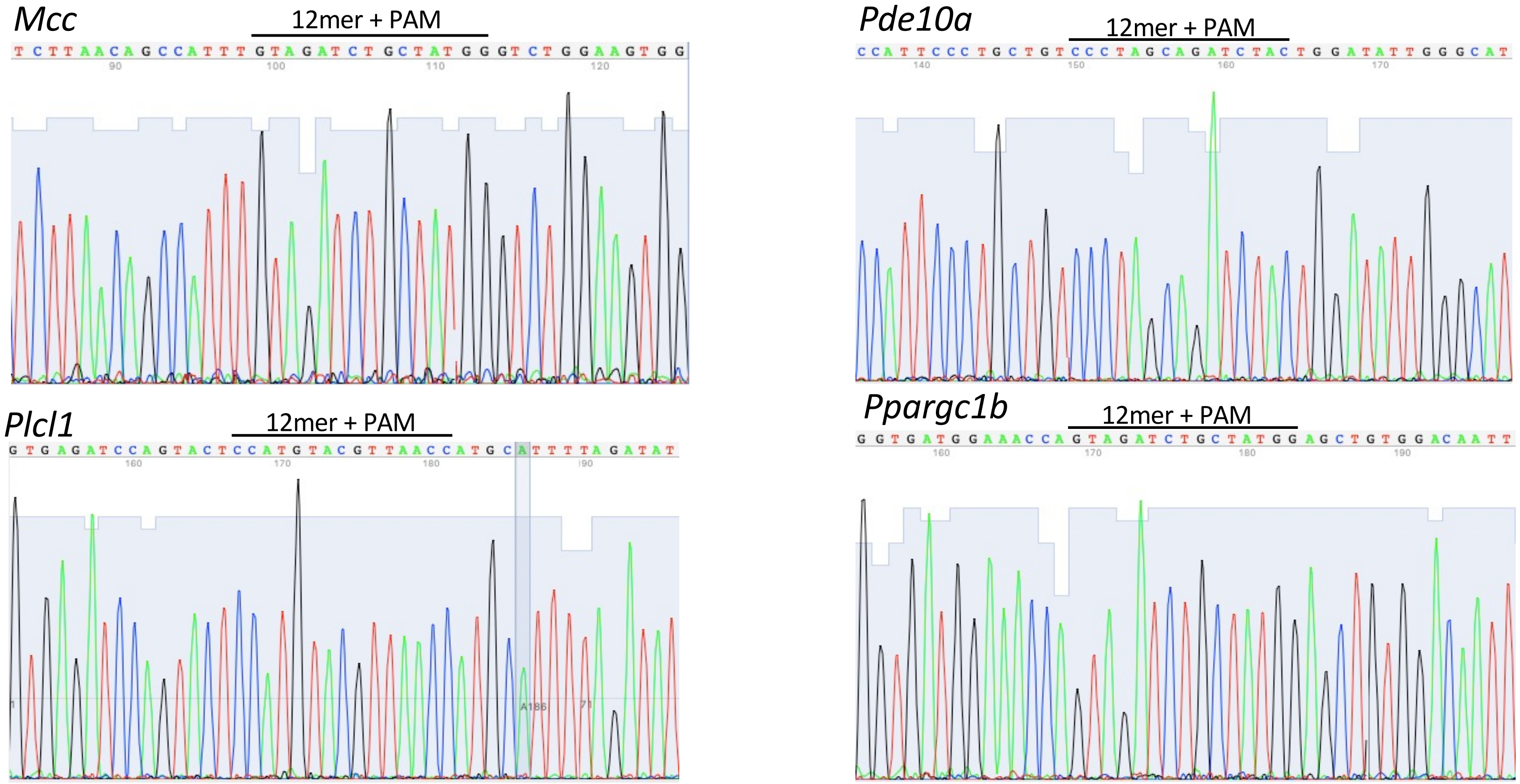

Supplement: S3 Fig — (A) Amino acid sequences deduced from the DNA sequences shown in Fig 1D. (B) Off-targeting candidate genes, which have a similar sequence to 12 nucleotides plus PAM targeted for the Cysltr1 gene. No off-target effects were found in the Cysltr1 KO mouse genome. (PDF) [file pone.0277307.s003.pdf]
