# Supplementary material for: Cysteinyl leukotriene receptor 1 is dispensable for osteoclast differentiation and bone resorption
Source: PLoS One. 2022 Nov 17;17(11):e0277307. doi: 10.1371/journal.pone.0277307 (PMC9671454; doi:10.1371/journal.pone.0277307)

S4 Fig

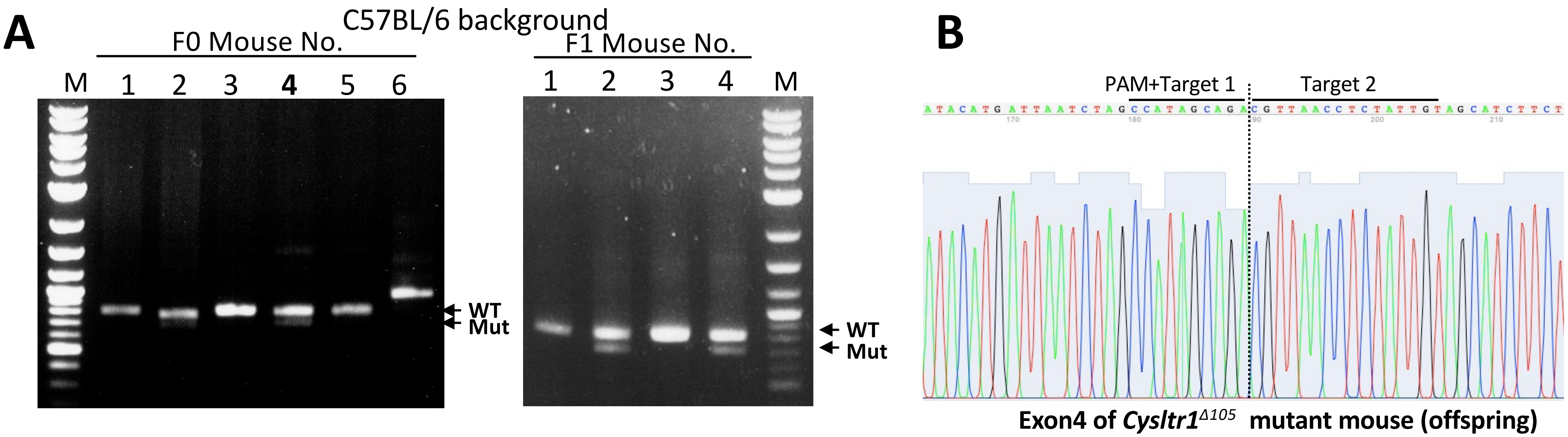

C

	mCysltr1 gene DNA sequence	Deletion size
WT	TAATCTAGCCATAGCAGATCTACTCTGTGTATGTACATTG(70bp)CCTATGCCTTGTACGTTAACCTCTATTGTAGCATCTTC	0 bp
Cysltr1$^{\Delta 105}$	TAATCTAGCCATAGCAGA-------------------------//---------------CGTTAACCTCTATTGTAGCATCTTC	105 bp

PAM + Target sequence

D

	CysLT1R amino acid sequence (C57BL/6 background)	Number of AA
WT	MYLQGTKQTFLENMNGTENLTTSLINNTCHDTIDEFRNQVYSTMYSVISVVGFFGNSFVLYVLIKTYHEKSAFQVYMINLAIAD<br>LLCVCTLPLRVVYYVHKGKWLFGDFLCRLTTYALYVNLYCSIFFMTAMSFFRCVAIVFPVQNINLVTQKKARFVCIGIWIFVIL<br>TSSPFLMYKSYQDEKNNTKCFEPPQNNQAKKYVLILHYVSLFFGFIIPFVTIIVCYTMIILTLLKNTMKKNMPSRRKAIGMIIV<br>VTAAFLVSFMPYHIQRTIHLHLLHSETRPCDSVLRMQKSVVITLSLAASNCCFDPLLYFFSGGNFRRRLSTFRKHSLSSMTYVP<br>KKKASLPEKGEEICNE	352
Cysltr1$^{\Delta 105}$	MYLQGTKQTFLENMNGTENLTTSLINNTCHDTIDEFRNQVYSTMYSVISVVGFFGNSFVLYVLIKTYHEKSAFQVYMINLAIAD<br>-----------------------------------VNLYCSIFFMTAMSFFRCVAIVFPVQNINLVTQKKARFVCIGIWIFVIL<br>TSSPFLMYKSYQDEKNNTKCFEPPQNNQAKKYVLILHYVSLFFGFIIPFVTIIVCYTMIILTLLKNTMKKNMPSRRKAIGMIIV<br>VTAAFLVSFMPYHIQRTIHLHLLHSETRPCDSVLRMQKSVVITLSLAASNCCFDPLLYFFSGGNFRRRLSTFRKHSLSSMTYVP<br>KKKASLPEKGEEICNE	317

S4 Fig (continued)

E

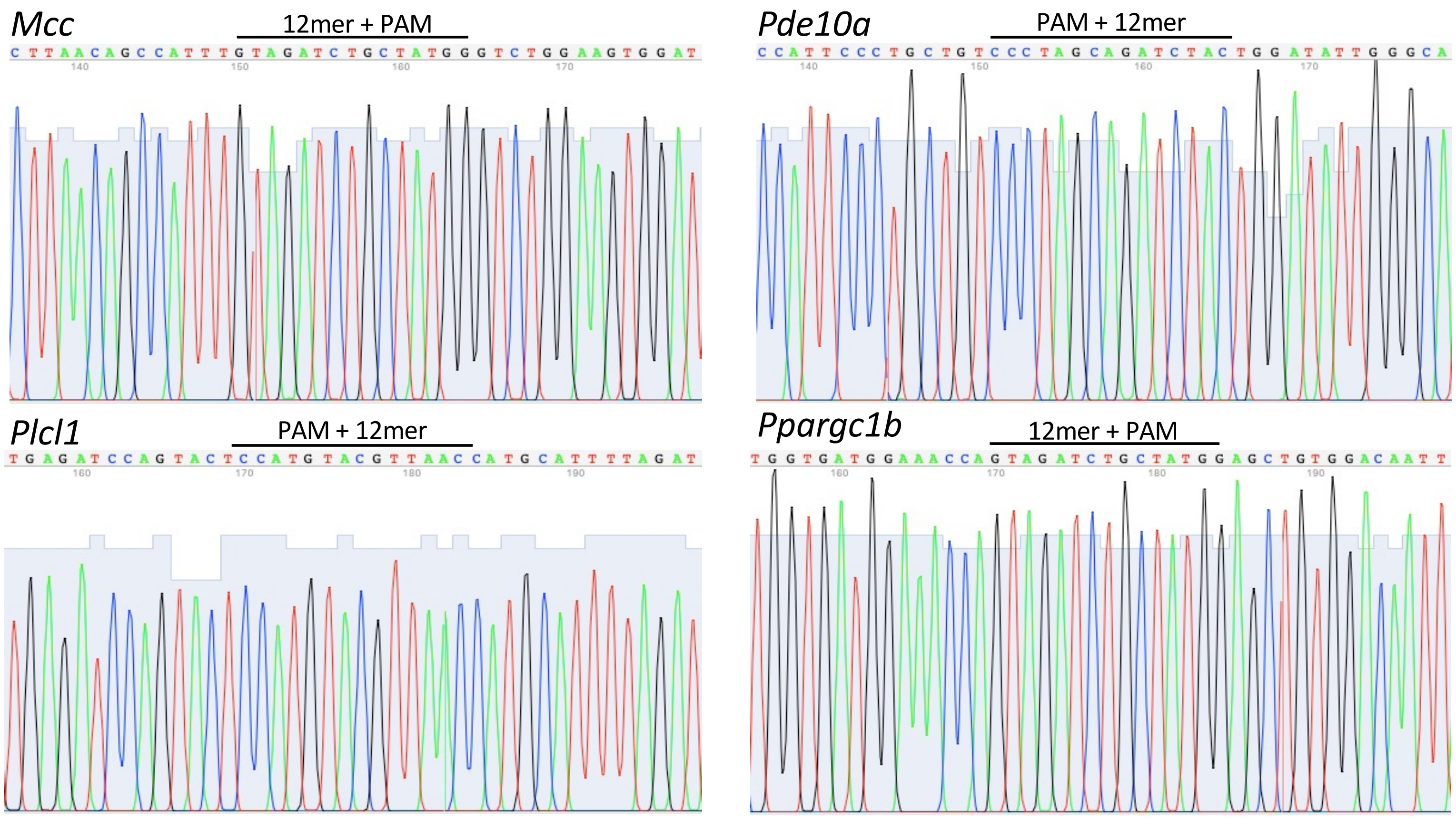

Supplement: S4 Fig — (A) Genotyping PCR of F0 (left) and F1 (right) pups after Cysltr1 gene editing in C57BL/6. The amplicon size for the wild-type (WT) Cysltr1 gene was 818 bp and that for the mutated gene was 713 bp. The #4 F0 female mouse was mated to a WT stud, providing the Cysltr1Δ105 strain (F1 mice). Sequencing chromatogram (B) and DNA sequence (C) of the mutation site in Cysltr1Δ105. The deletion size of the mutation site was 105 bp. (D) Deduced amino acid sequences of CysLTR1. (E) Sequencing chromatograms of the off-targeting candidate genes with 12-mer of the Cysltr1 target sequences adjacent to the PAM. No off-targeting was found in the Cysltr1 mutant mouse genome. “-” indicates deleted nucleotide bases. (PDF) [file pone.0277307.s004.pdf]
