# Supplementary material for: Cysteinyl leukotriene receptor 1 is dispensable for osteoclast differentiation and bone resorption
Source: PLoS One. 2022 Nov 17;17(11):e0277307. doi: 10.1371/journal.pone.0277307 (PMC9671454; doi:10.1371/journal.pone.0277307)

Fig 1C Raw gel images

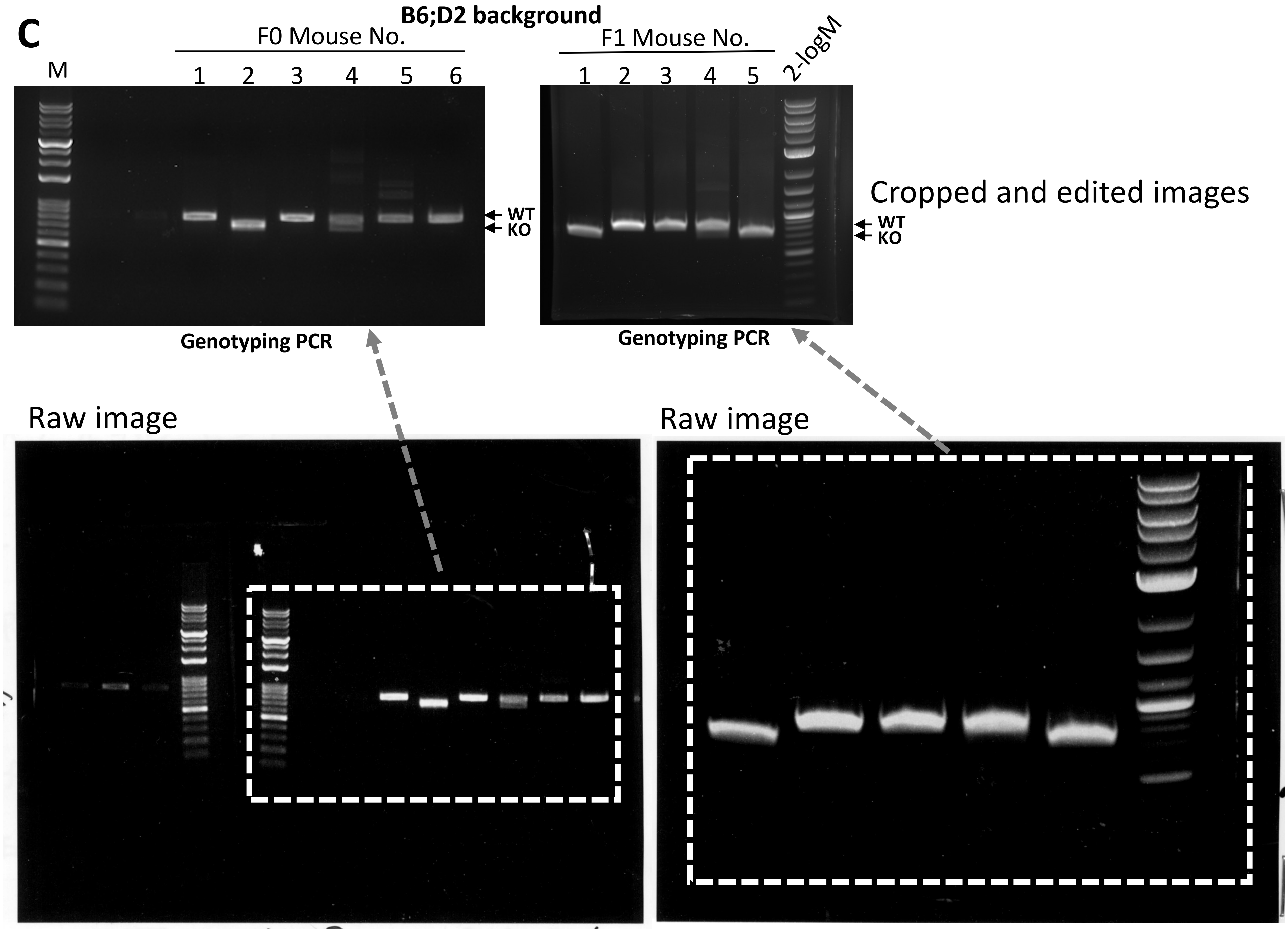

S4A Fig. Raw gel images

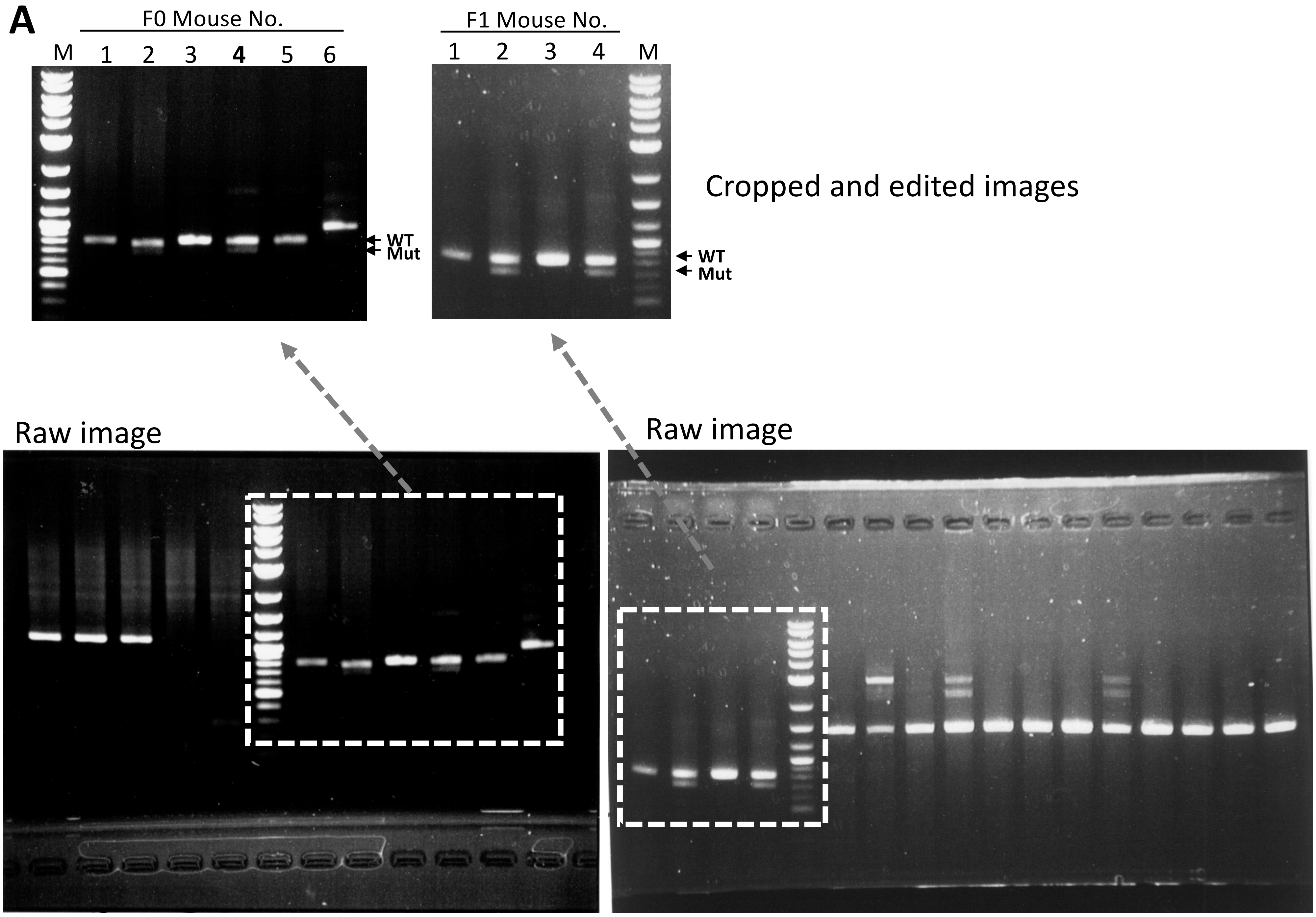

Supplement: S1 Raw images — (PDF) [file pone.0277307.s007.pdf]
