# Supplementary material for: Cysteinyl leukotriene receptor 1 is dispensable for osteoclast differentiation and bone resorption
Source: PLoS One. 2022 Nov 17;17(11):e0277307. doi: 10.1371/journal.pone.0277307 (PMC9671454; doi:10.1371/journal.pone.0277307)
Supplement: S1 Table — (PDF) [file pone.0277307.s009.pdf]

S1 table

Gene name		Sequences	Amplicon size
Cysltr1 (for genotyping)	Forward	GAGAACATGAATGGAACTGAAAATC	818 bp (WT)<br>706 bp (KO)<br>713 bp (*Cysltr1*$^{\Delta105}$)
	Reverse	GAATCACAGGGTCTAGTTTCACTGT	
Cysltr1 (for qPCR)	Forward	GGTCTATTATGTTCACAAAGGCAAG	195 bp
	Reverse	AATGTTCTGGACTGGAAAGACAAT	
Cysltr2 (for qPCR)	Forward	TTGAACTCCAAAGGACATATAATGG	200 bp
	Reverse	TACTGATCAAACATTTGCCGTACT	
Lpar4 (for qPCR)	Forward	GTTGCCAGTTTACACGTTTATTAGC	154 bp
	Reverse	CACCAGATTTATCTGAGACTTGGA	
Lpar6 (for qPCR)	Forward	CCTCTTCTTCCTGTGATGTCATATT	228 bp
	Reverse	TCGGGTACTTCTTCCTTCTTTTTAT	
P2ry10 (for qPCR)	Forward	GCCCAGAGTTGTACCTTTTTACTCT	182 bp
	Reverse	CATCACATCCTTCATACTGCTATTG	
