# Supplementary material for: Cysteinyl leukotriene receptor 1 is dispensable for osteoclast differentiation and bone resorption
Source: PLoS One. 2022 Nov 17;17(11):e0277307. doi: 10.1371/journal.pone.0277307 (PMC9671454; doi:10.1371/journal.pone.0277307)
Supplement: Attachment — Submitted filename: Response to Reviewers.docx [file pone.0277307.s010.docx]

**Response to reviewers**

**Reviewer #1:**

**General comment**

In the study by Fujita et al. the authors create a novel global mouse knockout model of the cysteinyl leukotriene receptor 1 (CysTR1). The authors analyze the skeletal phenotype of these mice. Unfortunately, the authors demonstrate that CyslTR1 is not necessary for osteoclast differentiation. The loss of CysTR1 does not lead to changes in the skeleton under pathological conditions. The authors do demonstrate successfully loss of Cysltr1 expression by recombination and that other related genes are not changed in expression due to the knockout of Cysltr1. There are some minor concerns that should be addressed and are listed below.

**Comment 1.**

In Figure 4 the images in panel A should be redone as there is no contrast and it is difficult to see the TRAP positive cells.

**Answer to comment 1**
We thank the reviewer #1 for the kind remarks. According to your advice, we replaced the previous images with high magnification ones in Fig 4A of the revised manuscript.

**Comment 2.**

Besides counting number in panel 4D the authors should also count size of the osteoclasts.

**Answer to comment 2**

According to your advice, we measured size of osteoclasts using ImageJ software. The data showed that Montelukast decreased osteoclast size in WT and *Cystlr1^Δ105^* BMMs. There was no significance in osteoclast size between WT and *Cystlr1^Δ105^* osteoclasts treated with Montelukast although we observed a tendency of suppression of Montelukast-decreased osteoclast size in *Cystlr1^Δ105^.* We added these results and sentences to the revised manuscript; the Fig 4F; Results section lines 381-385 and lines 402-403 in the revised manuscript with track changes.

**Comment 3.**

Should be stated in the text of the manuscript why the authors chose not to analyze the skeleton under physiological conditions.

**Answer to Comment 3.**

In response to the suggestion, we added the data of bone morphological analysis under physiological conditions. The results of histomorphometry and CT analysis did not show any noticeable differences between WT and *Cystlr1* mutants. We added these results and sentences to revised manuscript; the S6 Fig; Results section lines 365 and 368; Supporting information section lines 642-649 in the revised manuscript with track changes.

**Reviewer #2:**

**General comment**

This paper is an interesting analysis of the role of cysteinyl leukotriene receptor 1 in osteoclastic bone resorption. Experimental results using cell culture systems and bone resorption-promoting mouse models clearly demonstrate that cysteinyl leukotriene receptor 1 is not directly involved in osteoclast differentiation and bone resorption.

**Answer to comment**

We thank the peer review of reviewer #2.

**Reviewer #3:**

**General comment**

The authors investigated roles of cysteinyl leukotriene receptor 1 (CysLTR1) in osteoclastogenesis. To clarify the role in vivo they generated two lines of CysLTR1 mutant mice. Macrophages from those mutant mice did not respond to the stimulation of LTD4. However, these mutants failed to impact osteoclastogenesis in in vivo and in vitro studies. The experiments seems to be well organized and performed, and their conclusion is based on solid data. However, there are some concerns in the present manuscript.

**Major points**
**Comment 1**.

Figure 4A…To clearly show no difference in osteoclast number between WT and KO, the quantitative data of osteoclast number are needed. In addition, scale bar is missing.

**Answer to Comment 1.**

We thank the reviewer #3 for the kind remarks. According to your advice, we added quantitative data of osteoclast number of WT, KO and *Cysltr1^Δ105^* femur although the sample size was not sufficient as we would like to meet the dead line for revision. Histomorphometric analysis did not show any noticeable differences between WT and *Cystlr1* mutants. We added scale bars in Fig 4A. We added these results and sentences to the revised manuscript; S6 A-D Figs; Materials and methods section lines 201-203; Results section line 390; Supporting information section lines 642-645 in the revised manuscript with track changes.

**Comment 2**.

The authors only focused on the roles of CysLTR1 in osteoclast differentiation. To confirm whether the deficiency of CysLTR1 impacts on osteoclastic bone resorbing activity, serum bone resorption parameter such as serum CTX should be analyzed.

**Answer to Comment 2.**

As you pointed out, our study had lacked an analysis of the bone resorbing activity of osteoclasts. We performed pit formation assay for bone resorbing activity of osteoclasts differentiated from WT and *Cysltr1^Δ105^* BMMs using the osteoassay plate. The data showed that there was no difference in bone resorption activity *in vitro* between WT and *Cysltr1^Δ105^* osteoclasts. We added this result and sentences to the revised manuscript; the Fig 4D; the Materials and methods section lines 201-218; Results section lines 372-375, lines 387-388 and lines 396-398 in the revised manuscript with track changes.

**Comment 3.**

The explanation of roles of CysLT in bone resorption seems to be too short in Introduction. The authors should explain why they focus on CysLT in bone metabolism and roles of CysLT in bone metabolism and bone diseases in more detail.

**Answer to Comment 3.**

Thank you for your valuable suggestions. According to your advice, we explained roles of CysLTs in osteoclast differentiation and bone resorption, describing that CysLTs stimulate isolated osteoclasts to form resorption pits on calcified matrices and stimulate bone resorption in organ culture of mouse calvariae. In addition, LTD_4_ stimulates osteoclast differentiation from macrophage cell line RAW264.7 in the presence of RANKL. Previous our study showed that the CysLT synthesis substrate, GSH stimulated osteoclast differentiation. We added these sentences to the Revised Manuscript; Introduction section lines 71-77 and lines 80-81 in the Revised Manuscript with Track Changes. And we removed sentences from next paragraph line 84 in the revised manuscript with track changes.

**Comment 4**.

Fig. 6…Scale bars are missing in panels A and D.

**Answer to Comment 4.**

We added scale bar in Fig 6A and D. We added a sentence to the revised manuscript; Results section line 437 and line 443 in the revised manuscript with track changes.
